# Barriers and facilitators of physical activity in children with bronchiectasis: Perspectives from children and parents

**DOI:** 10.3389/fped.2022.974363

**Published:** 2022-10-05

**Authors:** Taryn Jones, Emmah Baque, Kerry-Ann F. O’Grady, Vikas Goyal, Anne B. Chang, Stewart G. Trost

**Affiliations:** ^1^School of Exercise and Nutrition Sciences, Queensland University of Technology, Brisbane, QLD, Australia; ^2^School of Health Sciences and Social Work, Griffith University, Brisbane, QLD, Australia; ^3^Australian Centre for Health Services Innovation, Queensland University of Technology, Kelvin Grove, QLD, Australia; ^4^Department of Respiratory and Sleep Medicine, Queensland Children’s Hospital, Brisbane, QLD, Australia; ^5^Gold Coast University Hospital, Gold Coast, QLD, Australia; ^6^Faculty of Medicine, The University of Queensland, Brisbane, QLD, Australia; ^7^Child Health Division, Menzies School of Health Research, Darwin, NT, Australia; ^8^School of Human Movement and Nutrition Sciences, The University of Queensland, Brisbane, QLD, Australia

**Keywords:** bronchiectasis, pediatrics–children, exercise, theoretical domains framework (TDF), qualitative analysis

## Abstract

**Background:**

Current bronchiectasis management guidelines recommend regular physical activity but a large proportion of children with bronchiectasis do not meet public health recommendations which call for 60 min or more of moderate-to-vigorous intensity physical activity daily. Knowing the factors that influence physical activity in children with bronchiectasis is necessary for the development of effective interventions to increase physical activity in this patient group. The objective of this study was to identify facilitators and barriers to physical activity in children with bronchiectasis unrelated to cystic fibrosis (CF) from the perspectives of children and their parents.

**Materials and methods:**

This was a qualitative study informed by the theoretical domains framework (TDF). Children aged 7–15 years (8.8 years, 8.4–11.0) (median, interquartile range) and parents (45.8 years, 39.7–48.3) completed separate, semi-structured interviews (*n* = 21). Recordings were transcribed verbatim, and barriers and facilitators related to each TDF domain deductively coded. Emergent themes were inductively derived via consensus moderation.

**Results:**

From the perspectives of children, fun with friends, organized sport and activities, and family co-participation in physical activity emerged as facilitators. Inability to keep up with their peers and time on technology emerged as barriers. From the perspectives of parents, instrumental and logistic support for physical activity and supportive social and physical activity environments emerged as facilitators, while management of symptoms associated with bronchiectasis emerged as a barrier.

**Conclusion:**

Programs to increase physical activity in children with bronchiectasis should be fun, accessible, provide opportunities for social interaction and address barriers related to exercise tolerance, perceived competence, and presence of respiratory symptoms.

## Introduction

Bronchiectasis is a chronic lung disease with abnormal peripheral airway dilatation objectively documented on a chest computed tomography scan (1, 2). Globally, over the last two decades the prevalence of bronchiectasis is increasing and recognized as an important cause of chronic respiratory disease and morbidity in children and adults (3, 4). The disease process alters the mucociliary clearance that contributes to persistent infection, airway obstruction and progressive lung damage if not managed early and effectively (5). Children with bronchiectasis present with a persistent wet cough, shortness of breath and poor exercise tolerance (1, 6). Living with bronchiectasis symptoms impacts the daily lives of children including schooling, play, physical activity, and general wellbeing (7, 8). The long-term impacts of bronchiectasis include higher prevalence of chronic diseases including depression, anxiety, cardiac dysfunction, in addition to poorer quality of life (QoL) outcomes (8–12). Current bronchiectasis treatments include pharmacological agents and airway clearance techniques along with general recommendations to maintain a healthy diet and regular physical activity (13).

There is strong and consistent evidence in support of the beneficial effects of regular physical activity on cardiovascular fitness and musculoskeletal health in children (14, 15). Physical activity also improves airway clearance (16), one mainstay treatment of people with bronchiectasis (13, 17). Further, there is evidence to conclude that physical activity can positively impact mental health, in addition to the positive impacts on blood pressure, adiposity and cardiovascular risk factors such as inflammatory biomarkers (14). Furthermore, children who accrue 60 min of moderate to vigorous physical activity (MVPA) each day have a lower risk of developing chronic diseases such as type II diabetes and metabolic syndrome later in adulthood (18).

Current guidelines for the treatment and management of bronchiectasis recommend regular physical activity (19, 20). Yet, the available evidence suggests that a large proportion of children with bronchiectasis do not meet public health recommendations for physical activity and that interventions to promote regular physical activity are needed (21, 22). In a recent cross-sectional study, Joschtel et al. (21) recruited 46 children with bronchiectasis [mean age ± standard deviation (SD) 7.5 ± 2.6 years] and objectively measured physical activity over 7 days. An accelerometer was used to determine daily time spent in sedentary, light, and MVPA. As a percentage of waking hours, participants were sedentary for 57.5% of the time, in light-intensity physical activity for 38.5% of the time and in MVPA for just 6.7% of the time. From this study, only two participants (5.6%) met the daily 60-min MVPA recommendation as opposed to 42% of healthy children in the normative comparison group.

Currently, there is a large body of research exploring barriers and facilitators to physical activity in the typically developing pediatric population (23–25). Sterdt et al. (24) conducted a systematic review of reviews on the correlates of physical activity in children and adolescents. Sex (male), age, ethnicity, parental education, family income, and socioeconomic status and parent education emerged as demographic and biological factors consistently associated with physical activity. Perceived competence, self-efficacy perceptions, goal orientation, and perceived barriers emerged as psychological, cognitive and emotional factors consistently associated with physical activity. Previous physical activity and participation in community sports emerged as behavioral attributes and skills consistently associated with physical activity. Parental support and support from significant others emerged as social and cultural factors consistently associated with physical activity, while access to recreational facilities, programs, and time outdoors emerged as physical environmental factors consistently associated with physical activity.

Fewer studies have examined barriers and facilitators in children with chronic health conditions. Pianosi and Davis (26) examined selected correlates of physical activity in children with asthma. Positive self-perceptions of motor skills and attitudes toward physical activity were identified as facilitators to participation in physical activity, while being affected by overweight or obesity was identified as a barrier. In a literature review focused on children with chronic respiratory conditions, Denford et al. (27) completed a synthesis of qualitative work exploring the barriers and facilitators to physical activity in children and young people with cystic fibrosis (CF). From the seven studies reviewed, nine themes were identified perceptions of physical activity, value attributed to physical activity, social influences, competing priorities, fluctuating health, normality, control beliefs, coping strategies and availability of facilities.

While there is evidence for healthy children and children with asthma and CF, to date, no studies have examined barriers and facilitators of physical activity in children with non-CF bronchiectasis. Given the important differences between children with CF and those without (1), bronchiectasis-specific data are required. Therefore, in this study we aimed to identify facilitators and barriers to physical activity in children with bronchiectasis from the perspectives of the parents and children.

## Materials and methods

### Participants

Participants for this study were recruited from respiratory clinics at the Queensland Children’s Hospital (QCH) or Gold Coast University Hospital (GCUH). The QCH and GCUH are large tertiary public hospitals that offer free or low-cost services as part of Australian public health system. Before or after their scheduled appointment, a member of the research team (TJ) discussed the study requirements with families to gauge interest and screen for eligibility. To be eligible, children (1) had a confirmed bronchiectasis diagnosis not related to CF; (2) were aged 6–15 years; and (3) were being managed by a respiratory physician. Families expressing an interest in the study were given a participant information sheet and consent form to consider their participation in the study. Prior to participation in the study, written informed consent was obtained from parents/guardians. Ethical approval for this study was received by the Queensland Children’s Hospital Human Research Ethics Committee (LNR/19/QCHQ/54135).

### Theoretical framework

The theoretical domains framework (TDF) informed the development of the interview guides for parents and children. The TDF is designed to assist researchers and health practitioners understand health behavior change, design interventions, and assess implementation problems (28, 29). The framework was developed and validated by Michie et al. (28) through a robust consensus process with a multi-disciplinary group of behavioral and implementation science researchers. This group identified 128 theoretical constructs from 33 existing behavior change and psychological frameworks relevant to implementation science to include 14 theoretical domains in the TDF.

### Interview guide

Separate interview guides were developed for children (see Table 1) and parents (see Table 2) based on selected domains of the TDF. For example, the question, “Do you feel that you are able to play and join in physical activity in the same way as your friends?” reflected the “Beliefs about Capabilities” domain of the TDF. Similarly, the question to parents “Do you feel your child has the skills needed to play with their peers?” reflected the “Skills” domain of the TDF. Prior to recruiting participants, the interview guide was piloted with three parents and two children not included in the study. Following the pilot, the ordering of some questions was modified, and additional conversational prompts included. The pilot interviews were not included for analysis. The parent interview guide included questions that related to the domains of knowledge, skills, social/professional role and identity, beliefs about capabilities, optimism, beliefs about consequences, reinforcement, intensions, environmental context and resources, social influences and emotion. The child interview guide included questions that related to the domains of knowledge, skills, social/professional role and identity, beliefs about capabilities, optimism, beliefs about consequences, reinforcement, intensions, social influences and emotion.

**TABLE 1:** Interview guide developed for children with associated TDF domain name.

TDF domain	Questions and prompts
Emotion	Can you tell me in your words what it is like having bronchiectasis?
Intentions	Outside of school hours how do you like to spend your time? Before school After school Weekends
Social role and identity	What type of extra-curricular activities to you do at the moment (not just sport)? What about at other times of the year? How did you choose these activities? What makes you enjoy this? What makes it not so fun?
Social influences	What activities do you enjoy doing with your family?
Social role and identity	During PE at school what parts do you like? Being on the oval, playing on the courts, practicing different skills, dancing.
Social influences	During lunch break or PE do you feel you comfortable playing games and joining in physical activity with your friends and classmates?
Beliefs about capabilities	Do you feel that you are able to play and join in physical activity in the same way as your friends? Does having bronchiectasis change anything?
Skills	What do you see makes it tricky to join in or enjoy sport and physical activity?
Beliefs about capabilities	What would help you to join in or enjoy sport and physical activity?
Beliefs about consequences	Can you think of time when you haven’t been able to play or join in sports and games due to bronchiectasis? Can you tell me about this? How did this make you feel (did it make symptoms different)?
Social influences	How you do feel when you are playing sport and joining in physical activity? How do you feel if you don’t get to join in on sport and play?
Optimism	Do you feel that as you get older you will get better at different sports and games?
Reinforcement	If you practice a new activity does it get easier or harder for you? What is a game or sport that you find really hard? What makes it hard?
Skills	What activities do you find easy (include screen activities)? How did these get to be easy for you?
Knowledge	Do you feel that sport and play can change your bronchiectasis (or how your lungs work)?
Sweeping question	Are there comments you would like to add before we finish?

**TABLE 2:** Interview guide developed for parents with associated TDF domain name.

TDF domain	Questions and prompts
Emotion	Can you share how bronchiectasis has impacted your family and (child’s name) childhood?
Intentions	How does (child’s name) like to spend their spare time outside of school hours? • Before school • After school • Weekends
Intentions	What type of extra-curricular activities does (child’s name) participate in? • How often, what seasons, what other activities? • How did you choose these activities? (siblings, child’s interest, cost).
Social role and identity	I am interested in games and play as much as I am organized extra-curricular activities. Does your child enjoy sport, games or playing outside at home or example? • What types of games and play do they enjoy most? • Do you enjoy or feel comfortable joining in with your child?
Social influences	Do you feel they are able to play and join in physical activity in the same way as their peers?
Beliefs about capabilities	What do you see makes it tricky for (child’s name) to join in or enjoy sport and physical activity? What would help (child’s name) to join in or enjoy sport and physical activity? What help’s (child’s name) to join in or enjoy sport and physical activity?
Optimism	Do you feel as your child gets older they will enjoy more physical activity?
Skills	Do you feel your child has the skills needed to play with their peers? • That they will acquire more skills to join in more physical activity? • What has helped them have those skills? • What has made it harder for your child to acquire those skills?
Environmental context and resources	Does your community make it easy to spend time outside, (pathways, safe places to cross the road, parks)?
Reinforcement	Do you feel that physical activity and general playing can impact the symptoms of bronchiectasis for your child? Have you noticed this in the short term, for example after a big walk or a game with friends? • Over time, say a year, do you notice the impact of physical activity?
Beliefs about consequences	In times where you you’ve noticed less physical activity do you feel it impacts how you child feels of their health?
Knowledge	There are recommendations and guidelines for children around physical activity, have you come across these before? Do you feel parents of children with bronchiectasis would think it is helpful to know more about these guidelines?
Optimism	Do you feel optimistic that you can include more opportunities for play and physical activity with your child?
Sweeping question	Is there comments you would like to add before we finish?

### Data collection

Participants completed a single interview with a researcher (TJ) via videoconference (due to COVID-19 restrictions). Parent interviews occurred prior to child interviews. The parent was present during the child’s interview (either sitting with or nearby), but the questions were directed to the child. Interviews were digitally recorded and transcribed verbatim. Transcriptions were compared to the recording for accuracy, fully de-identified, and assigned a unique study identification number. Recruitment to the study continued until saturation had been reached (i.e., no new information was offered by participants). Interviews were completed between August 2020 and July 2021.

### Data analysis

Qualitative data from the interviews were analyzed using thematic analysis. The analysis was completed in two stages with a deductive then inductive approach. The first stage involved coding participant’s responses to questions in each TDF domain as a “barrier” or “facilitator.” Initially, two members of the research team (EB and TJ) read through three randomly selected transcripts (two child and one parent) line by line and coded responses as a “barrier” or “facilitator.” Barriers were responses that referred to sentiments that prevented or hindered children’s physical activity. Facilitators were responses that referred to views that increased or supported children’s physical activity. The researchers then compared their independent coding to verify agreement and resolve discrepancies. After agreement was verified, TJ read each transcript and coded responses as either a “barrier” or “facilitator.”

Stage two of data analysis involved separate thematic analyses of text coded as “barriers” or “facilitators” in stage one. An inductive approach was used to identify recurrent themes for children and parents using the six steps identified by Braun and Clarke (30) which include: becoming familiar with the interview, initial coding, generating themes, reviewing themes, defining the name of the themes, and writing up the analysis. After reading the transcripts TJ commenced initial coding. Initial codes were assigned based on the content of the transcript. For example, if a parent spoke about their backyard pool, this text would have two initial codes of “yard” and “swimming pool.” Throughout the initial coding phase, two members of the research team (EB and TJ) met to review the codes to ensure that the code names reflected the content of the transcripts and reach consensus on the codes. Once initial coding was complete, all members of the research team discussed initial code groupings based on the content and sentiments. Initial code groupings were then compared and aggregated to form subthemes. Differences in initial code grouping and subthemes were discussed until consensus was reached. From there, a process of consensus moderation was used to identify final emergent themes. Differences were discussed until consensus was reached and themes named. NVivo 12 (QSR International Pty. Ltd.) was used to manage and organize the textual data.

## Results

### Participant characteristics

From the 21 families approached in hospital clinics, 11 families consented (11 parents and 10 children). All parent respondents were female, with a median age (IQR) of 45.7 (39.7–48.3) years. Eleven children were scheduled for an interview and ten children completed interviews (one child was unable to participate due to ongoing illness). Children were aged from seven to 15 years (8.8 years, 8.8–11.0). Two of the 10 children were females. Parent interviews ranged from 21 to 54 min (mean duration 33 ± 10.1 min) and child interviews between 12 and 28 min (mean duration 20 ± 5.6 min). Socio-economic indexes for areas (SEIFA) is derived from Australia’s census data and ranks areas according to relative socio-economic advantage and disadvantage (31). SEIFA scores are transformed into deciles where one represents the most disadvantaged areas and then represents the most advantaged. In the current study participant’s SEIFA decile ranged from three to eight, so was well distributed across low to high areas of socioeconomic status. Families who participated in this study resided in postcodes that offered a variety of affordable and accessible activities and organized sports.

### Themes from the perspective of children

The TDF domain, initial codes, initial code groupings, subthemes, and final emergent themes related to children’s facilitators and barriers to physical activity are shown in Tables 3, 4, respectively. From the perspectives of children, fun with friends, organized sport and activities, and family co-participation in physical activity emerged as facilitators. Difficulty of keeping up with their peers emerged as the primary barrier. Below, individual themes are presented with illustrative quotes from participants.

**TABLE 3:** Development of themes relating to facilitators for physical activity from the perspective of children.

TDF domain	Initial codes	Initial code groupings	Sub-themes	Emergent themes
Emotion	Other activities, symptoms of bronchiectasis not impacting, knowledge	Fun Fun describing activity Fun (with friends or family)		
Intentions	Organized activities, ability to join in, activities with family, parent, friends, sibling, other activities, swimming, football, joining in with friends, commute to and from school, tennis, feeling about being with friends, basketball, fun (with friends or family), musical instrument		Fun	Fun with friends
Social role and identity	Organized activity, ability to join in, swimming, friends, fun, joining in with friends, fun describing activity, other activity, skills to join in, fun (with friends or family), feeling about being with friends, tennis, activities with family, football, knowledge about bronchiectasis, cricket, basketball, musical instrument, commute to and from school	Friends Joining in with friends Ability to join in Feeling about being with friends Activities with friends Friends feelings on symptoms	Friends
Social influences	Friends, activities with family, ability to join in, organized activity, siblings, parents, feeling about being with friends, swimming, joining in with friends, friends feeling on symptoms, other family members, dance, footfall, fun, tennis, ability to keep up	Swimming Football Tennis Basketball Cricket Other activities	Organized sport	Organized sports and activities
Beliefs about capabilities	Symptoms of bronchiectasis not impacting, ability to join in, activities with family, activities with friends, friends’ feelings on symptoms			
Skills	Fun, friends, ability to join in, fun (with friends or family), fun describing activity, ability to join in, knowledge, organized activity, swimming, coughing, activities with family, parent	Dance Musical instrument Other activities Organized activities	Activities	
Beliefs about consequences	Knowledge about bronchiectasis, ability to join in, feelings about bronchiectasis			
Optimism	Ability to join in, knowledge, symptoms of bronchiectasis not impacting, organized activity			
Reinforcement	Ability to join in, organized activity, feeling optimistic, knowledge on bronchiectasis, friends, ability to join in, symptoms of bronchiectasis not impacting	Ability to join in Activities with family Parent Sibling Other family members	Participating in activities with family	Family co-participation physical activity
Knowledge	symptoms of bronchiectasis not impacting, organized activity, swimming, friends, ability to join in, activities with family, parent		Family members	

TDF domains are presented in the same order as the interview guide.

**TABLE 4:** Development of themes relating to barriers for physical activity from the perspective of children.

TDF domain	Initial codes	Initial code groupings	Sub-themes	Emergent themes
Emotion	Symptoms of bronchiectasis, location of symptoms, feelings about bronchiectasis, fatigue, coughing			
Intentions	Screen time (tablet), screen time (gaming), commute to and from school, Screen time (television), screen time (PC) organized activities, symptoms of bronchiectasis, friends, activities with family, siblings, no organized activity, fun	Lack of ability Lack of ability to keep up Lack of skills Other responsibilities No organized activity (Difficulty) joining in with friends (Difficulty)joining in/playing with friends	Lack of ability	
Social role and identify	Lack of ability, symptoms of bronchiectasis, organized activity, fatigue, breathlessness, fun, lack of ability to keep up, screen time (gaming), screen time (PC), other activities, fun with friends, fun with family, activities with family, no organized activity			Difficulty keeping up with peers
Social influences	Friends, screen time (gaming), feeling about being with friends, (difficulty) joining in/playing with friends, lack of ability to keep up, lack of ability, feelings about bronchiectasis, organized activity, symptoms of bronchiectasis, friends feelings on symptoms, activities with family			
Beliefs about capabilities	Lack of ability to keep up, feelings about bronchiectasis, coughing, fatigue, breathlessness, location of symptoms, friends	Symptoms of bronchiectasis Location of symptoms Feelings about bronchiectasis Fatigue Coughing Shortness of breath Friends feelings on symptoms	Symptoms of bronchiectasis	
Skills	Lack of skills, Symptoms of bronchiectasis, lack of ability, lack of ability to keep up, screen time, screen time (television), Screen time (gaming), friends, coughing, breathlessness, fatigue, fun, knowledge, organized activity, location of symptoms, friends feelings on symptoms, (difficulty) joining in with friends,			
Beliefs about consequences	Feelings about bronchiectasis, symptoms of bronchiectasis, Lack of ability to keep up, friends, lack of ability, friends, knowledge, breathlessness, location of symptoms, coughing	Screen time (tablet) Screen time (gaming) Screen time (television) Screen time (gaming)	Screen time	Time on technology
Optimism	Lack of time, other responsibilities			
Reinforcement	Lack of ability to keep up, lack of ability			
Knowledge	Symptoms of bronchiectasis, lack of knowledge, lack of ability, coughing			

TDF domains are presented in the same order as the interview guide.

#### Child facilitator: Fun with friends

When discussing physical activity and games and play, children consistently spoke about having fun with their friends. Children reported they liked to play games with their friends, whether it be organized sport, school lunch time play or playground visits. They valued being included by their friends.

*“I really like playing soccer, because I want to play soccer and I have my best friend that she normally comes over and we go down to the track*…*I like to go out and I like doing it with all my friends.” Ch04*

*“My friends get me to go up and make sure I am involved with the game.” Ch01*

#### Child facilitator: Organized sport and activities

Most children talked about participation in organized sport and activities across the year. There were a large variety of activities with swimming and football [football (known as soccer in Australia), rugby league and Australian Rules Football] the most frequently reported. Common organized activities included dance and musical instruments.

*“I played soccer, a bit of swimming, umm all different sports but soccer’s kinda the main one.” Ch06*

“…*in dancing I do ballet, tap, jazz, jazz funk*… *I’m going to start contemporary. I also play football but, I’m changing to boxing next year.” Ch11*

#### Child facilitator: Family co-participation in physical activity

Children spoke about their family and the activities they do together. Children spoke about their siblings and how they spend their time trampolining, bike riding and swimming being the most frequently mentioned activities.

*“I play with (sister), we muck around with each other, and I play with (brother)*… *we bounce on the tramp” Ch04*

*“Sometimes me and my brother just go riding around the street.” Ch05*

“…*we get to do swimming with my mum and sister” Ch11*

Children also spoke about time spent with parents for less structured activities such a bike riding, swimming and backyard ball games.

“…*I hop on my bike and we run around, I ride around and mum follows me around” Ch07*

*“so basically, um, it’s really fun. Sometimes dad will take us to the park. Other times we’ll do a scooter. Sometimes we’ll catch up with some friends.” Ch08*

*“Yeah. I find it easy to go out and kick the footy with dad some afternoons.” Ch01*

#### Child barrier: Difficulty keeping up with peers

Children discussed how it was difficult for them to keep up with their friends when playing sports and doing physical activity. Children expressed the challenges in joining games or play as they perceived themselves to be slower at running and that they needed to practice new skills and activities more than their peers. All children spoke about how coughing and having difficulty breathing during exercise was a barrier to physical activity.

“…*it was hard cause basically, I just stand there while everyone else was kicking goals and stuff.” Ch07*

*“like when I am running, my lungs get puffed out a lot*… *like I have to stop and take five breaths, then I get tackled” Ch02*

*“But, I just like give up over time*…*I’ll be playing and sometimes I have to sit down for a rest, sometimes because I get pretty worn out*…*” Ch05*

“…*it makes me stop, cough. It makes me stop, wait a minute, and then I have to cough and then catch my breath and then go back on learning for like 2 min and then do it and then do it again*… *I just stand there and watch people do stuff*…*because I keep on stopping a lot and losing my friends” Ch07*

#### Child barrier: Time on technology

Children spoke about screen time across a range of devices including home computers, televisions, tablets and phones. Screen time was discussed across many questions irrespective of TDF domain and occasionally brought up without a question being asked.

“…*running you have to do actual more fitness where Xbox you’re just sitting on your couch” Ch05*

“…*Sometimes my friend will come over, we mainly only play Terraria together because they were, Yeah, but Ah, and because they love playing on the old Xboxes, are usually on the when you have the best stuff and everything.” Ch07*

*“Play on my iPad. I watch TV and stuff.” Ch10*

*“I play my iPad until it dies.” Ch02*

### Themes from the perspective of parents

The TDF domain, initial codes, initial code groupings, subthemes, and final emergent themes related to children’s facilitators and barriers to physical activity are shown in Tables 5, 6, respectively. Logistic and instrumental support and supportive social and physical activity environments emerged as facilitators, while management of symptoms associated with bronchiectasis emerged as the primary barrier. Individual themes are presented with illustrative quotes from participants.

**TABLE 5:** Development of themes relating to facilitators for physical activity from the perspective of parents.

TDF domain	Initial codes	Initial code groupings	Sub-themes	Themes
Emotion	Lack of symptoms of bronchiectasis, organized activities, swimming, soccer, family support	Swimming Soccer AFL Dance Basketball	Organized sport and activities	
Intentions	Resources, equipment, built environment, family support, organized activity, swimming, other organized activity, bike/bike riding, trampoline, yard (backyard), park (playground, green space), beach, dance, lack of symptoms of bronchiectasis, commute to/from school, soccer, screen time, friends, football, hockey, Australian football league, COVID			
Social role and identity	Family support, equipment, yard, park, lack of symptoms of bronchiectasis, knowledge, joining in with friends, organized activity, tennis	Trampoline Bike riding Park Beach Yard Tennis Playing with friends Other organized activity		Parental instrumental and logistic support
Social influences	Ability to join in, family support, joining in with friends, knowledge, resources, equipment, organized activity, Australian Football league, skills to join in, lack of symptoms of bronchiectasis		Organization of family activities	
Beliefs about capabilities	Playing with friends, support from family, organized activities, ability to join in, knowledge, lack of symptoms of bronchiectasis, lack of cough			
Skills	Family support, ability to join in, joining in with friends, lack of symptoms with bronchiectasis, resources, equipment, park	Swimming Tennis	Built environment	
Environmental context and resources	Resources, equipment, built environment, family support, organized activities, football, swimming, friends, basketball			
Reinforcement	Knowledge, family support, resources, equipment, ability to join in, organized activity, swimming, soccer, lack of symptoms of bronchiectasis	Beach Park (playground, green space) Yard (backyard)	Natural environment	
Beliefs about consequences	Knowledge, family support, organized activities			
Knowledge	Knowledge, family support, park, bike/bike riding	Equipment Bike/bike riding Trampoline	Equipment	Supportive social and physical activity environments
Optimism	Family support, knowledge, organized activity, equipment, ability to join in			

TDF domains are presented in the same order as the interview guide.

**TABLE 6:** Development of themes relating to barriers for physical activity from the perspective of parents.

TDF domain	Initial codes	Initial code groupings	Sub-themes	Themes
Emotion	Symptoms of bronchiectasis, lack of initial diagnosis, lack of family support, fatigue, lack of ability to keep up, lack of knowledge, work commitments, COVID, unable to join in (friends), cough, feelings about bronchiectasis	Symptoms of bronchiectasis Fatigue Cough breathlessness		
Intentions	Screen time, symptoms of bronchiectasis, lack of time, fatigue, COVID, lack of ability, lack of organized activity, sibling (family)		
Social role and identity	Lack of initial diagnosis, symptoms of bronchiectasis, cough, lack of ability, lack of ability to keep up, screen time		Presence of symptoms	
Social influences	Symptoms of bronchiectasis, lack of ability, fatigue, unable to join in (friends), cough, lack of skills, lack of ability to keep up, lack of knowledge			
Beliefs about capabilities	Symptoms of bronchiectasis, lack of ability, unable to join in (friends), lack of ability to keep up, fatigue, lack of time, screentime, couch			Management of symptoms associated with bronchiectasis
Skills	Lack of skills, lack of ability to keep up, symptoms of bronchiectasis, breathlessness, lack of ability, fatigue, cough			
Environmental context and resources	Lack of initial diagnosis, distance from friends	Lack of ability Lack of skills Unable to join in (friends) Lack of ability to keep up	Inability to keep up	
Reinforcement	Symptoms of bronchiectasis, lack of initial diagnosis, lack of knowledge, family, friends, fatigue, peer influence			
Beliefs about consequences	Symptoms of BE, screen time, lack of knowledge, cost of organized activity		
Knowledge	Lack of knowledge, lack of initial diagnosis, symptoms of bronchiectasis, lack of family support			
Optimism	Symptoms of bronchiectasis, COVID, peer influence, lack of ability to keep up, lack of initial diagnosis, fatigue, lack of knowledge, screen time			

TDF domains are presented in the same order as the interview guide.

#### Parent facilitator: Parental logistic and instrumental support

Similar to the children, parents described their child’s involvement in a variety of organized sports and activities (swimming being the most popular) and gave examples of how they routinely organized family outings and activities that provided children with physical activity such as games at home like handball, trampolining and going to playgrounds, parks and beaches.

“… *3 days a week would be with soccer. He was doing swimming squad there for a while as well, so that was early mornings and the occasional afternoon as well.” Par06*

*“Activity-wise, we try to get out a lot. I take them to the park. He rides his scooter. He’s learning how to ride a bike.” Par02*

*“It hasn’t been hard to say, hey, let’s go down for a bike ride. Let’s go to the skate park and he’s happy to spend 2 or 3 h there easily*…*we play tennis, as a family. We don’t play competition tennis or anything like that. No, I mean tennis wise we will go down and spend, you know, half our time just hitting with him and throwing balls*… *but now he plays quite well with us” Par01*

#### Parent facilitator: Supportive social and physical activity environments

Parents identified having the right equipment and supportive physical activity environments as facilitators to their child’s physical activity. Parents spoke about the equipment and resources available either at home or locally that supported physical activity. Trampolines, bikes, and basic sporting equipment likes balls and racquets were commonly reported to be available at home. Parents not only spoke about access to parks and nature areas in their community but also the size of their backyards and the presence of footpaths to promote walking.

“…*in the summer he’s in the pool and in the winter, he’s, I’ve just bought him another trampoline*… *The trampoline has been amazing, because especially when he is sick, he goes out there and it helps him cough and get rid of bit of the rubbish.” Par05*

*“There’s a little park up the road we might go to that, he’ll go bike riding out on the street with these friends or things we might go find a new park. He loves to climb. So, generally we stick to like the climbing tree park or anything that’s got something on it for him to climb.” Par07*

“…*we don’t have a huge backyard but we’ve got enough*…*and we’ve got the park down the road to play football*…*yeah, he climbs trees when he plays with the kid next door.” Par02*

#### Parent barrier: Management of symptoms associated with bronchiectasis

Parents discussed the presence of bronchiectasis symptoms such as coughing, volume of mucus, shortness of breath, and fatigue as barriers to their child’s participation in physical activity.

*“He’s slow with his running, because of the coughing. At the time, we didn’t know, it was–we just knew that it was mucus, and it was making it difficult for him to–because he would always stop and cough up all the phlegm.” Par02*

*“He gets worn out probably quicker. He doesn’t seem to have as much dexterity or strength as they do*… *He tends to fatigue really quickly compared to them. That’s probably the most pronounced difference” Par09*

“…*when he does get that little cough and he starts to get the gunk he knows or my chest is hurting, that’s generally a sign that we’ve got to take it easy, take a step back*… *He’s quite wiped out by the time we do finish school. So, at the moment, he just sort of comes home, he has a snack.*… *but sometimes he’ll get out and he’ll ride a bike or play with his friends outside but during, as the week progresses, he gets quite tired pretty quickly.” Par07*

## Discussion

This study is the first to investigate the facilitators and barriers to participation in physical activity in children with bronchiectasis. Children and their parents presented unique perspectives and distinct themes relating to barriers and facilitators to participation in physical activity. Facilitators to physical activity from the perspectives of children included family co-participation in physical activity, having fun with friends and participating in a variety of organized sport and activities. Children identified an inability to keep up with their peers and screen time as barriers to physical activity. Supportive social and physical activity environments and parental logistic and instrumental support were facilitators to physical activity according to patents and the management of symptoms associated with bronchiectasis a key barrier.

Having fun with friends was identified by the children as a key facilitator to physical activity and was discussed throughout the interviews across multiple TDF domains. Similar sentiments of “enjoyment,” “fun,” “peers,” and “friends” were expressed in qualitative studies of children with CF (32, 33). In a recent study of children with CF, it was reported that friends and friendship groups could positively influence physical activity (34). A multi-disciplinary team of health practitioners, including respiratory physicians, nurses, physiotherapists, and exercise physiologists, are involved in the management of bronchiectasis and well positioned to advocate strategies to increase physical activity. Our findings suggest that health professionals can support children with bronchiectasis by assisting families to identify physical activity options that include peers and are, by nature, enjoyable. Whenever possible, therapeutic exercise programs for children with bronchiectasis could involve peers or siblings and include active games that are developmentally appropriate and fun.

Participation in organized sport and activities was also identified as a facilitator to physical activity. Children discussed their participation in organized activities and sports throughout the interviews and in response to multiple questions from different TDF domains. There is consistent evidence from qualitative studies involving children with chronic health conditions that supports the role of organized sport and other recreational activities in promoting regular physical activity (35, 36). Just as pharmacological agents and airway clearance techniques are prescribed for children with bronchiectasis; organized sport and active recreational activities can be recommended as a strategy to promote physical activity. When appropriate, health professionals should encourage children to try new organized sports and activities if previous experiences have not been as successful or engaging as hoped.

Parent support and co-participation was another key facilitator of children’s physical activity. Children explicitly spoke about playing active games, sport, and other activities with parents and caregivers. This finding in consistent with the results of previous studies investigating parental influences on physical activity in children with CF, asthma, and other chronic health conditions (27, 35). A parent’s belief that physical activity will positively impact symptoms, along with their motivation and due diligence in planning physical activity experiences for their children were examples of parental supports described in a study of children with asthma, CF and type I diabetes (35). In a systematic review that included 35 children with CF, Denford et al. (27) found parental support in the form of planning, structuring and providing physical activity opportunities for their children to be important influences on children’s participation in physical activity. Collectively, these findings highlight the need for parents and caregivers to be educated about the benefits of participating in physical activity with their child and the importance of providing the logistic and instrumental support required for ongoing participation in physical activity, sport, and physically active recreational pursuits.

The inability to keep up with peers was identified as the predominant barrier to participation in physical activity, with the presence of bronchiectasis symptoms and lack of motor skill proficiency key contributors. Children spoke about their breathlessness, coughing and feeling fatigued when participating in physical activity with their peers. These observations are consistent with results of studies involving children with CF. In those studies, it was common for children with CF to acknowledge that their symptoms, such as tiredness or breathlessness could limit participation in physical activity (32, 34). In addition to poor exercise tolerance, children repeatedly spoke about their lack of skills or their inability to play games and sports with their peers. This finding is consistent with the results of a recent study examining the relationship between fundamental movement skill proficiency and physical activity in children with bronchiectasis (37). In that study, fewer than 5% of children demonstrated mastery of locomotor skills such as run, gallop, hop, and leap; while fewer than 10% demonstrated mastery of object control skills such as two-handed strike, overarm throw, and underarm throw. Only 17.4% of children with bronchiectasis achieved their age equivalency for locomotor skills and just 8.7% achieved their age equivalency for object control skills (37). Importantly, children achieving their age equivalency for fundamental movement skills exhibited significantly higher levels of MVPA than children not achieving their age equivalency (51.7 vs. 36.7 min/day). Collectively these findings highlight the need for exercise programs to address poor exercise tolerance and fundamental movement skill proficiency for this patient group.

Screen-based digital technology such as smartphones, iPads, video games, computers and television were discussed by children as a barrier to physical activity. Children found it easier to spend time on screens than participating in physical activity. The negative impact of excessive screentime on child health is well documented (38–40). Whilst it is unknown if children with chronic respiratory conditions such as CF, asthma and bronchiectasis spend more time on screens than their healthy peers, it has been identified that only a small percentage of children with bronchiectasis are sufficiently active for health benefit (21). Health professionals can promote autonomy supportive parenting strategies that manage or limit screen time. For example, a family media plan encourages families to reflect on family values, consider the purpose of screen time, and create appropriate goals and rules (41, 42). Parents offered informed perspectives in relation to the barriers and facilitators to physical activity. Parental logistic and instrumental support for physical activity emerged as a key facilitator. This theme was consistent with children’s perspectives on parental support and co-participation as a facilitator of physical activity. Parents talked about signing up, paying fees, providing transport before and after school, and organizing family activities that promoted physical activity. Similar findings regarding the role of parental support for physical activity have been reported for children with CF (35, 43). Parents of children with cerebral palsy similarly identified the importance of logistic and instrumental support for physical activity in terms of advocacy, motivation and communication with trainers and coaches (44). These findings suggest that health professionals should encourage parents and caregivers of children with bronchiectasis to set goals for their child’s participation in physical activity.

Parents identified a supportive built environment and support from extended family and friends and as key facilitators. Parents discussed the value of having basic sporting equipment such as balls and racquets at home, along with bikes, trampolines, large backyards, and in some cases, swimming pools. In a unique perspective, parents also identified walkable neighborhoods and easy access to local parks and nature areas as important positive influences on their child’s physical activity behavior. The influence of the built environment on physical activity among children with chronic respiratory conditions has received little research attention. Happ et al. (43) reported that having access to a bicycle as part of a home-based exercise program was a facilitator to physical activity among children with CF. In this study social environments included parents, extended family, siblings, friends, and neighbors. Extended family members such as grandparents provide logistic support to enable physical activity in addition to motivation and encouragement. Friends and siblings support physical activity via active play at home and in local parks, playgrounds and sporting fields. Health professionals can provide information about local parks and facilities in addition to programs or activities that support physical activity.

For parents, the management of symptoms associated with bronchiectasis was the key barrier to physical activity. They described how symptoms such as coughing, breathlessness, vomiting and fatigue negatively prevented their child from participating in physical activity. Identical findings have been reported for parents of children with asthma and CF (27, 32, 35). Children with bronchiectasis may have ongoing symptoms that impact their daily lives including schooling, play, physical activity, and general wellbeing (6). While physical activity may be limited during periods of exacerbation when children are unwell, exercise may in fact play an important role in managing symptoms and preventing future declines in lung function. In a study of adults with bronchiectasis, an 8-week therapeutic exercise program significantly reduced the number of exacerbations over a 12-month period (45). Further evidence focusing on the potential benefits of physical activity and the health impacts of therapeutic exercise programs is needed for children with bronchiectasis.

This study is unique in the pediatric bronchiectasis literature, includes children and parents as participants and includes only those with current diagnoses. Participants were recruited from two specialist pediatric pulmonology clinics in south-east Queensland, Australia, and may not reflect other demographics. It would be appropriate for future studies to include participants from diverse geographical areas and clinical settings to establish if the findings are consistent. The perspectives of health professionals where not gathered for this study. Future studies could include health professionals as participants to gather their perspectives on barriers and facilitators to physical activity in children with bronchiectasis.

In summary, the current study provided insights into the factors that influence physical activity behavior in children with bronchiectasis. Having fun with friends, participating in organized sport and activities, and family co-participation in physical activity were facilitators from the perspectives of children. Inability to keep up with their peers and time on technology emerged as barriers. From the perspectives of parents, instrumental and logistic support for physical activity and supportive social and physical activity environments emerged as facilitators, while management of symptoms associated with bronchiectasis emerged as a barrier. Programs to increase physical activity in children with bronchiectasis should therefore be fun, accessible, and provide opportunities for social interaction. Therapists involved in the delivery of physical activity programs should consider poor exercise tolerance, low perceived competence, developmental delays in motor skill proficiency, and the management of symptoms. Presently, there are limited community options which provide these opportunities. The findings of this study should inform the design and implementation of therapeutic exercise programs for children with bronchiectasis.

## Data availability statement

The original contributions presented in the study are included in the article/supplementary material, further inquiries can be directed to the corresponding author.

## Ethics statement

The studies involving human participants were reviewed and approved by Queensland Children’s Hospital. Written informed consent to participate in this study was provided by the participants’ legal guardian/next of kin.

## Author contributions

ST oversaw the conduct of the study. AC, VG, and TJ supported the recruitment. TJ interviewed the participants, reviewed the transcripts and coded, and drafted the manuscript. ST, EB, K-AO’G, and TJ analyzed the data. ST, EB, K-AO’G, AC, and VG reviewed the manuscript. All authors had a role in informing the conceptual framework used in coding and analysis of the data collected, read, and approved the final manuscript.
